# Case Report: Burden of Illness in Narcolepsy Type 1: Hikikomori in a Teenage Girl

**DOI:** 10.3389/fpsyg.2021.634941

**Published:** 2021-03-12

**Authors:** Marco Filardi, Vincenza Blunda, Stefano Vandi, Alessandro Musetti, Annio Posar, Paola Visconti, Fabio Pizza, Giuseppe Plazzi, Christian Franceschini

**Affiliations:** ^1^Department of Biomedical and Neuromotor Sciences (DIBINEM), University of Bologna, Bologna, Italy; ^2^Istituto Di Ricovero e Cura a Carattere Scientifico, Istituto delle Scienze Neurologiche di Bologna, Unità Operativa Semplice d'Istituto Disturbi dello Spettro Autistico, Bologna, Italy; ^3^Istituto Di Ricovero e Cura a Carattere Scientifico, Istituto delle Scienze Neurologiche di Bologna, Unita Operativa Complessa Clinica Neurologica, Bologna, Italy; ^4^Department of Humanities, Social Sciences and Cultural Industries, University of Parma, Parma, Italy; ^5^Department of Biomedical, Metabolic and Neural Sciences, University of Modena and Reggio Emilia, Modena, Italy; ^6^Department of Medicine and Surgery, University of Parma, Parma, Italy

**Keywords:** narcolepsy, hikikomori (social withdrawal), actigraphy, delayed sleep phase, adolescent, sodium oxybate

## Abstract

Narcolepsy type 1 (NT1) deeply impacts on quality of life, especially during adolescence, with NT1 children and adolescents that frequently report difficulties in integration with peers and decreased participation in after-school activities. Here we describe the case of NT1 teenager girl presenting with severe physical and social withdrawal, fulfilling the proposed diagnostic criteria for hikikomori, together with the classic NT1 symptoms. Social withdrawal is an overlooked phenomenon among NT1 children and adolescents that, if present, require a multidisciplinary approach and personalized interventions, but patients can benefit from NT1 pharmacological treatment.

## Introduction

Narcolepsy type 1 (NT1) is a rare neurological disorder characterized by excessive daytime sleepiness (EDS), untimely REM sleep manifestations, and nocturnal sleep disruption (Plazzi et al., 2018). NT1 mostly arises during childhood/early adolescence and is frequently accompanied by a rapid weight gain up to obesity (Ponziani et al., 2016). Moreover, in addition to the classic symptoms, pediatric NT1 patients are at increased risk of impairment in several cognitive domains and frequently exhibit emotional and externalizing problems (Plazzi et al., 2018).

Since the late 90s, a severe condition of prolonged social withdrawal, also known by the Japanese term “hikikomori,” has been described among Japanese teenagers and young adults (Teo, 2010). The term hikikomori describes a phenomenon characterized by a severe form of physical and social withdrawal: individuals with hikikomori isolate themselves in their own home, stop going to school or the workplace, and refuse interactions with peers and people outside the family unit (Kato et al., 2019). Initially considered a Japanese culture-bound phenomenon, hikikomori cases have been described in several countries (Malagón-Amor et al., 2015; Teo et al., 2015).

Although not included in the current DSM, a set of diagnostic criteria for hikikomori have been proposed (Teo and Gaw, 2010). To meet these criteria, the subject must (1) stay at home or in their room for most of the day, almost all days; (2) avoid social participation (stopping going to school or the workplace) and social relationships with peers and family members; (3) experience significant functional impairment or distress associated with social isolation; and (4) continuous social isolation has to last 6 months or more. Hikikomori syndrome has a high comorbidity with anxiety, mood disorders (Koyama et al., 2010) and Internet addiction (Kato et al., 2020) and has been associated with wet beriberi (Tanabe et al., 2018) and elephantiasis nostras verrucosa (Moriuchi et al., 2015). Here we present a case of NT1 associated with pathological social withdrawal. The case report has been conducted according to the CARE guidelines (Riley et al., 2017).

## Case Description

A 15-year-old girl, adopted at the age of 3 after spending 1 year in community, at the age of 11 started to present severe EDS during the school hours and resumed the habit of napping in the afternoon. Nocturnal sleep became interrupted by frequent awakenings; she also experienced episodes of sudden muscular weakness triggered by laughter/anger and an abrupt weight gain. At the age of 12, she was diagnosed with a specific learning disorder.

At the age of 13, she was admitted to our Narcolepsy Center (December 2018).

**Figure 1:**
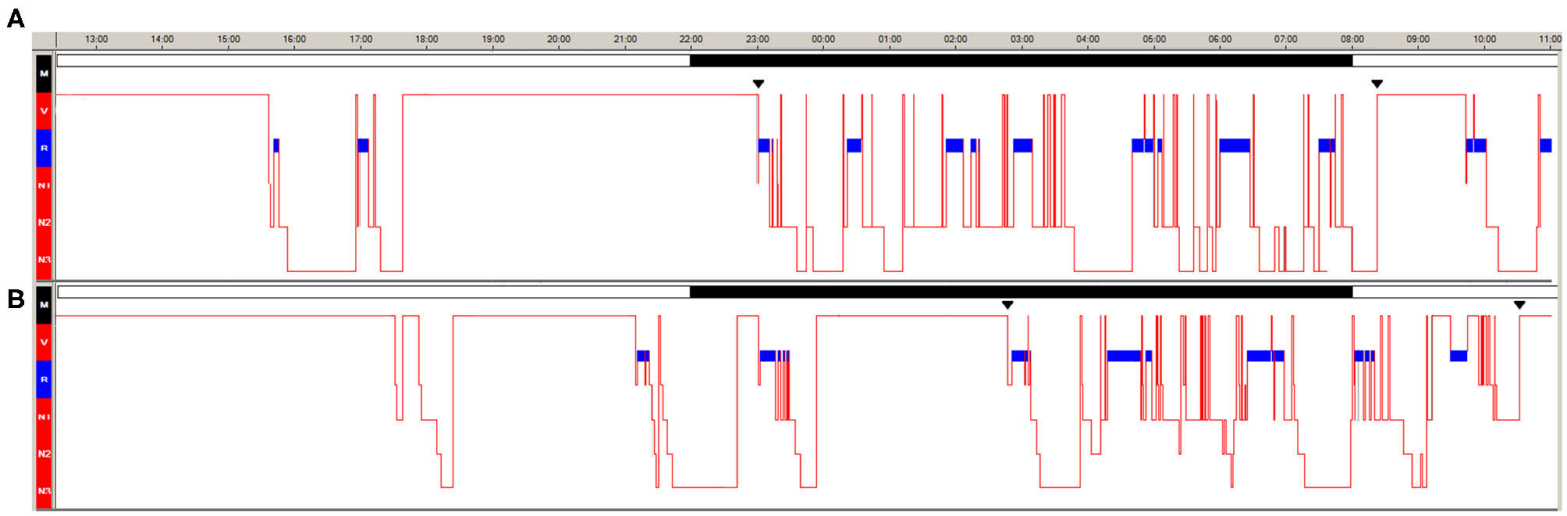
Twenty-four hours v-PSG during the first **(A)** and second **(B)** hospitalization. The blue bar indicates REM sleep. Continuous black bars indicate the ward's lights-off and lights-on times.

Clinical, neurological examination and brain MRI were normal, but the patient was overweight (BMI: 25.7) and markedly sleepy (Epworth Sleepiness Scale for Children and Adolescents, ESS-CHAD = 18/24). Psychiatric evaluation performed by a psychiatrist with vast experience in child and adolescent psychiatry (A.P.) did not disclose personality disorders or depressive mood (Children's Depression Inventory 2, CDI 2: 1/54). Twenty-four-hour video-polysomnography (v-PSG) documented two sleep episodes (one in the early morning and one in the afternoon) characterized by direct transition into REM sleep (sleep onset REM periods, SOREMP) (Figure 1A) and the multiple sleep latency test documented pathological sleep latency (3.12 min with 4/5 SOREMP). She denied hypnagogic/hypnopompic hallucinations and sleep paralysis; cataplexy was video-documented through a standardized procedure. The patient carried the HLA DQB1^*^06:02 allele and had reduced cerebrospinal fluid hypocretin-1 level (82.47 pg/mL).

Accordingly, NT1 was diagnosed and a treatment with Pitolisant up to 36 mg daily started with a remarkable improvement of EDS and cataplexy.

After 7 months of physical and mental well-being, during which the patient regularly attended school and sport activity, she reported loss of efficacy of the treatment with Pitolisant (June 2019).

Subsequently (July 2019), she quit sport activity and voluntarily withdrew from social life, rarely leaving her parents' home (less than once a month) and spending most of the day on the Internet and playing video games. At the start of the new school year (September 2019), she sporadically attended school for about a month and definitely dropped out of school in October.

Shortly thereafter, she further isolated herself rarely leaving her room and refusing to communicate even with her parents and to leave the room for having lunch/dinner (her mother left the meals outside the door). Moreover, she started to delay the onset of the sleep phase.

**Figure 2:**
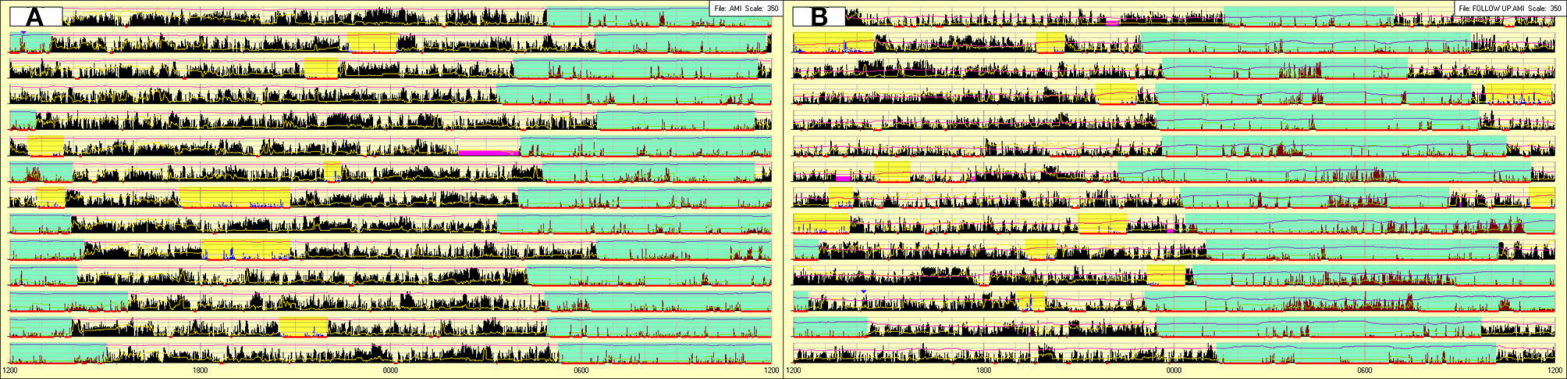
Actigraphy prior to the second hospitalization **(A)** and after 3 months of stable treatment with sodium oxybate **(B)**. Sky-blue highlight = nocturnal sleep period. Yellow highlight = diurnal sleep episodes. Fuchsia highlight = periods of device removal.

In December 2019, she underwent a second hospitalization, preceded by actigraphy (Figure 2A). Clinical and neurological examinations were unremarkable, subjective sleepiness decreased (ESS-CHAD: 12/24) but her weight did not change (BMI: 26.18).

Psychiatric evaluation disclosed social withdrawal behavior, attentional deficits, impaired cognitive flexibility (NEPSY-II test battery), and a low emotional quotient (emotional quotient inventory–youth version score: 60, normal range 90–110) with relevant difficulties in several emotional domains including adaptability, general mood, and positive impression.

Actigraphy (Figure 2A) documented the typical profile of NT1 children (nocturnal hyperactivity and frequent diurnal naps) coupled with a delayed sleep phase (bedtime 4:48 ± 1:04, wake-up time: 13:15 ± 1:24) (Filardi et al., 2016).

The 24-h v-PSG documented several diurnal sleep episodes in the late afternoon and early evening (*n* = 5, 3/5 with SOREMP), delayed sleep onset (bedtime at 1:14), and reduced nocturnal total sleep time (Figure 1B).

Based on the presence of delayed sleep–wake phase and socially withdrawn behavior, a treatment with sodium oxybate up to 7 g daily (3.5 grams at about 23:30 and a second equal dose after 3.5 h) associated with individual psychotherapy was started.

After 3 months of stable pharmacological treatment (February 2020), the follow-up actigraphy (Figure 2B) documented a partial normalization of the sleep/wake schedules but also highlighted the presence of periods, concurrent with the second sodium oxybate intake, characterized by sudden increase of light and intense motor activity, indicating that during several nights the patient remained awake for prolonged time, most likely to play video games.

As expected, the treatment with sodium oxybate induced a major weight loss (Filardi et al., 2018) and the patient's BMI returned within the normality range (BMI: 20.44).

On the other hand, the psychotherapeutic intervention (delivered “in person”) has proven beneficial with the patient who was slowly starting to resume relations with the closest peer.

## Discussion

This is the first report of hikikomori in a patient with NT1.

**Figure 3:**
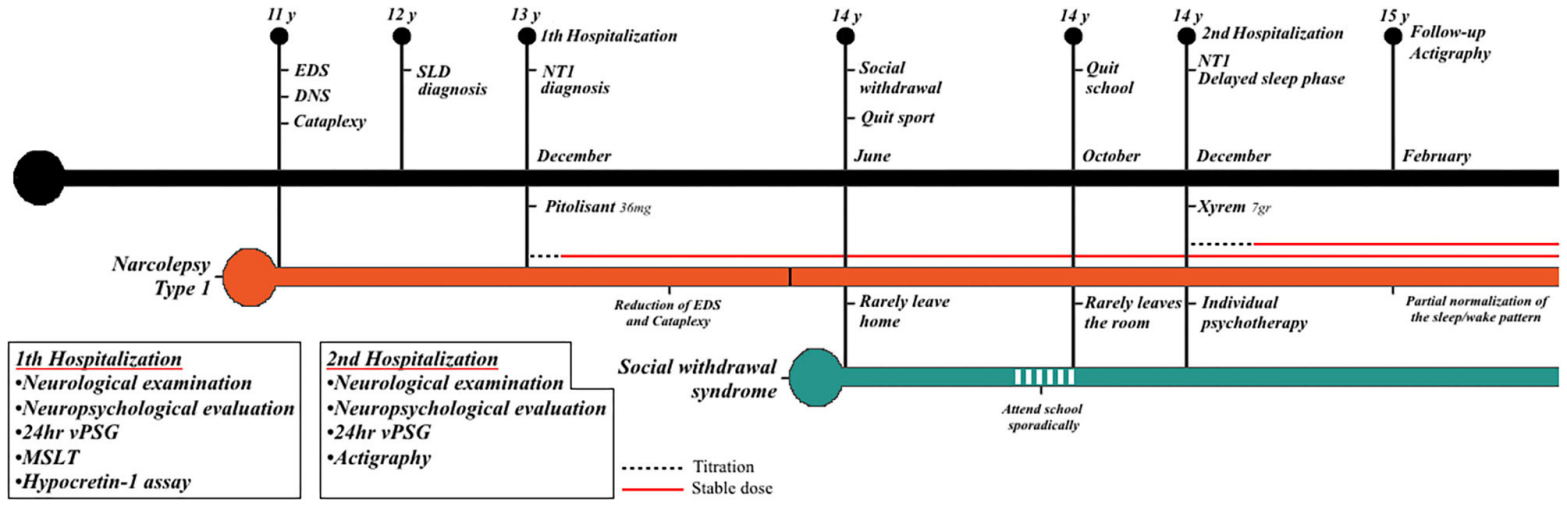
Timeline with relevant data from the episode of care and evaluations performed. Black bar = lifetime events. Orange bar = NT1 disease course. Cyan bar = social withdrawal. NT1, narcolepsy type 1; EDS, excessive daytime sleepiness; DNS, disrupted nighttime sleep; SLD, specific learning disorder; v-PSG, video-polysomnography; MSLT, multiple sleep latency test.

Our patient fulfilled the proposed diagnostic criteria for hikikomori, presenting with severe physical and social withdrawal that lasted for more than 6 months and significantly interfered with her functional life (school dropout) (Teo and Gaw, 2010). Nonetheless, the patient did not complain of distress associated with the social isolation; at the psychiatric evaluation, when asked about the reasons for her withdrawal behavior, she did not provide an explanation but firmly reaffirmed her intention of not wanting to go to school anymore and stay home all day. A timeline with relevant data and evaluations carried out are reported in Figure 3.

Although the etiology of hikikomori is still unknown, we speculate that several NT1 features may have facilitated the social withdrawal of our patient.

First, disrupted nighttime sleep is a common feature in NT1 children and adolescents who present numerous brief awakenings, often with difficulties returning asleep (Roth et al., 2013).

These long-lasting periods of wakefulness, when parents are asleep, represent a window of opportunity for online gaming and excessive Internet use, which in turn might have prompted the patient to delay the sleep phase in order to align it with the online gaming schedule.

Indeed, delayed sleep phase is rare in pediatric NT1 patients (Filardi et al., 2016), while social withdrawal syndrome has been associated with irregular sleep–wake pattern (Chauliac et al., 2017) and sleep–wake rhythm inversion has been described in hikikomori cases (Gondim et al., 2017).

Second, the rapid weight gain that frequently accompanies NT1 onset (Ponziani et al., 2016), as in our patient, drastically modifies physical appearance and consequently perceived body image.

Third, NT1 negatively impacts on quality of life, especially during adolescence, with several studies that have reported poor school performance with difficulties in integration, frequent absenteeism, and decreased participation in after-school activities (Plazzi et al., 2018).

Basing on studies that highlighted an association between peer-related loneliness and maladjustment, we can speculate that the patient's difficulties hamper the establishment of stable social relationships with peers and may have contributed to social withdrawal (Musetti et al., 2020).

Fourth, hikikomori subjects have a higher lifetime prevalence of anxiety disorders (Koyama et al., 2010) and depression (Teo, 2013) and are more likely to have autistic tendencies (Katsuki et al., 2020).

Similarly, NT1 children and adolescents have an increased susceptibility to psychiatric disorders (Blackwell et al., 2017) and cases of NT1 with comorbid autism spectrum disorder have been recently described (Prihodova et al., 2018).

In our case, the concomitant presence of NT1 symptoms and social withdrawal behavior significantly complicated the patient's pharmacological and behavioral management.

Indeed, in a relatively short period of time we had to modify the pharmacological therapy twice and refer the patient to psychotherapeutic treatment. Family support, home visit by social workers or psychologists, and individual psychotherapy are recommended and considered effective for hikikomori subjects (Kato et al., 2019) and similarly could prove beneficial for NT1 patients with concomitant hikikomori syndrome. A possible limitation of the present report is that the child and adolescent psychiatrist did not conduct a structured clinical interview to rule out the presence of psychiatric comorbidity. Social withdrawal may be an overlooked phenomenon among NT1 children and adolescents that, if present, requires multidisciplinary management. Actigraphy may prove useful to track the disease course over time and ecologically monitor treatment response and adherence.

## Data Availability Statement

The original contributions presented in the study are included in the article/supplementary material, further inquiries can be directed to the corresponding author/s.

## Ethics Statement

Ethical review and approval was not required for the study on human participants in accordance with the local legislation and institutional requirements. Written informed consent to participate in this study was provided by the participants' legal guardian/next of kin. Written informed consent was obtained from the minor(s)' legal guardian/next of kin for the publication of any potentially identifiable images or data included in this article.

## Author Contributions

All authors listed have made a substantial, direct and intellectual contribution to the work, and approved it for publication.

## Conflict of Interest

GP participated in the advisory board for UCB Pharma, Jazz Pharmaceuticals, Idorsia, and Bioprojet, outside the submitted work. The remaining authors declare that the research was conducted in the absence of any commercial or financial relationships that could be construed as a potential conflict of interest.

## Abbreviations

NT1: narcolepsy type 1
EDS: excessive daytime sleepiness
ESS-CHAD: Epworth Sleepiness Scale for Children and Adolescents
SOREMP: sleep onset REM period
BMI: body mass index
v-PSG: video-polysomnography
CDI 2: Children's Depression Inventory 2
DSM: Diagnostic and Statistical Manual of Mental Disorders.
